# Metabonomics reveals that entomopathogenic nematodes mediate tryptophan metabolites that kill host insects

**DOI:** 10.3389/fmicb.2022.1042145

**Published:** 2022-11-10

**Authors:** Yuan Zhang, Fang Wang, Zihua Zhao

**Affiliations:** ^1^Department of Plant Biosecurity, College of Plant Protection, China Agricultural University, Beijing, China; ^2^Institute of Plant Protection, Ningxia Academy of Agricultural and Forestry Sciences, Ningxia, China

**Keywords:** *Steinernema feltiae*, *Xenorhabdus bovienii*, Trp metabolism, 3-HAA, EPNs

## Abstract

The entomopathogenic nematode (EPN) *Steinernema feltiae*, which carries the symbiotic bacterium *Xenorhabdus bovienii* in its gut, is an important biocontrol agent. This EPN could produce a suite of complex metabolites and toxin proteins and lead to the death of host insects within 24–48 h. However, few studies have been performed on the key biomarkers released by EPNs to kill host insects. The objective of this study was to examine what substances produced by EPNs cause the death of host insects. We found that all densities of nematode suspensions exhibited insecticidal activities after hemocoelic injection into *Galleria mellonella* larvae. EPN infection 9 h later led to immunosuppression by activating insect esterase activity, but eventually, the host insect darkened and died. Before insect immunity was activated, we applied a high-resolution mass spectrometry-based metabolomics approach to determine the hemolymph of the wax moth *G. mellonella* infected by EPNs. The results indicated that the tryptophan (Trp) pathway of *G. mellonella* was significantly activated, and the contents of kynurenine (Kyn) and 3-hydroxyanthranilic acid (3-HAA) were markedly increased. Additionally, 3-HAA was highly toxic to *G. mellonella* and resulted in corrected mortalities of 62.50%. Tryptophan metabolites produced by EPNs are a potential marker to kill insects, opening up a novel line of inquiry into exploring the infestation mechanism of EPNs.

## Introduction

Biological interaction is one of the fundamental principles of ecosystems (Shi and Bode, 2018). Due to serious environmental pollution caused by pesticide abuse in recent years, many scientists have sought efficient and environmentally friendly insecticides to reduce environmental pollution, such as evaluating microbial control agents applied to the soil-inhabiting stages of pests (Ricci et al., 1996; Campos-Herrera et al., 2021; El Aimani et al., 2021). The entomopathogenic nematodes (EPNs) in the phylum Nematoda are obligate insect parasites, and the only free-living stage of the nematodes, the infective juvenile (IJ), carry symbiotic bacteria to infect many insects and can cause septicemia and death of host insects within 24–48 h (Akhurst, 1980; Ozakman and Eleftherianos, 2021). EPNs belonging to the families Heterorhabditidae and Steinernematidae are safe to humans and the environment and do not involve resistance build-up by target pests (Wakil et al., 2022). When insects are infested by EPNs, they mount a series of immune reactions to defend themselves. Normally, the host insect turns on some metabolic pathways to resist EPN infestation, while EPNs release toxic molecules to cause metabolic disorders of the host (Agus et al., 2018). For example, excessive accumulation of reactive oxygen species and Trp metabolites will cause disease and even death (Zhang et al., 2019; Leitner et al., 2022). However, biomarkers that cause insect death and impair insect immunity are produced by bacteria, such as fabclavine and some peptides (Elias et al., 2020; Wenski et al., 2020; Kenney et al., 2021). *G. mellonella* is an excellent model for research into the molecular mechanisms underlying host resistance to nematode infection, the pathogenicity of nematodes, and how nematodes and bacteria persist and reproduce within their hosts (San-Blas and Gowen, 2008; Monteiro et al., 2014; Booysen et al., 2022).

The circulatory system of insects is of the “open” type, in which the blood cavity is the entire body cavity, and all the internal organs are immersed in the blood (Guillott, 1980). The hemolymph of insects contains a variety of proteins, enzymes, and hormonal and lipid carrier proteins, which are not only related to tissue formation and material metabolism but also affect insect growth and development, the invasion of foreign substances, and other immune mechanisms (Blow and Douglas, 2019). Among them, esterases, a crucial class of proteins that can catalyze the hydrolysis of ester bonds, can cleave esters into acids versus alcohols *via* hydrolysis with the participation of water molecules (Wang et al., 2018). These esterases affect the transport and metabolism of normal lipids in insects and nerve conduction, participate in the detoxification process of a variety of drugs, environmental toxicants and carcinogens, protect insects from toxicants, and develop resistance (Lord and Potter, 1954; Bhatt et al., 2020). Enzymatic bioremediation is a potential and rapid method for toxin biodegradation (Sutherland et al., 2004; Bhatt et al., 2020). The changes in esterase activity in insects infected with nematodes and symbiotic bacteria can reflect the decomposition of lipids and then reflect the degree of destruction of the insect immune system (Pearce et al., 2017; Liu et al., 2021).

With further research, metabolomics has been widely introduced into the study of disease mechanisms, biomarkers, tissue development regulation, and other related molecular mechanisms (Chevrette et al., 2019; Modoux et al., 2020; Chen et al., 2022). Metabolomics is a comprehensive analysis of metabolites in specific biological systems using high throughput mass spectrometry and nuclear magnetic resonance, among which UPLC-HRMS is the most promising metabolomics analysis tool internationally recognized to date due to its high accuracy and resolution and the simultaneous analysis of multiple indicators (Kouba et al., 2022; Pan et al., 2022). Untargeted metabolomics combined with biological epigenetic observation is used to detect the dynamic changes of many metabolites before and after stimulation or disturbance, to find metabolites with different expressions, and then clarify the metabolic processes of organisms (Snyder et al., 2013; Bode and Challinor, 2015; Kim et al., 2017). Yu et al. (2017) isolated and purified four novel metabolites from liquid cultures of *X. bovienii* SN52 by extensive column chromatography and semipreparative HPLC and identified the abovementioned compounds as Trp derivatives with cytotoxicity to insects by comprehensive NMR spectra and HR-ESI-MS analysis. Moreover, Tse et al. (2017) found that 3-HAA acts as a precursor of kleboxymycin with high insecticidal activity.

Trp metabolism is the basic metabolic pathway in organisms; Trp is the most chemically complex amino acid and the best substrate for extensive transformation (Krautkramer et al., 2020), and xenocyloins and tryptamides synthesized by *Xenorhabdus* bacteria are derived from Trp (Li et al., 1993; Bode et al., 2017; Yu et al., 2017). There are three main pathways of Trp metabolism in insects: (1) Trp is converted into Kyn and its downstream products by indoleamine 2,3-dioxygenase 1 (IDO 1; Clarke et al., 2013). The downstream products of Kyn, such as 3-HAA, are closely related to many biological processes of insects and bacteria, such as neurotransmission, immune response, and disease (Tse et al., 2017; Gao et al., 2018). (2) Trp is converted to serotonin (5-hydroxytryptamine [5-HT]) by tryptophan hydroxylase 1 (TpH 1), which plays an important role in regulating physiological responses by activating serotonin receptors as an important signal component (Yano et al., 2015; Kim et al., 2017; Farrelly et al., 2019). (3) Intestinal symbiotic bacteria transform into indoles and their derivatives, including indoles, indole-3-acid-acetic (IAA) and indole-3-aldehyde (IAld), which are ligands of AhR (Zelante et al., 2013; Alexeev et al., 2018). AhR is a key component of the immune response at barrier sites and acts on immune cells, playing a crucial role in maintaining homeostasis (Agus et al., 2018; Lamas et al., 2018).

## Materials and methods

### Insects and EPNs

*Steinernema feltiae* and *Galleria mellonella* were preserved and bred at the Plant Quarantine and Invasion Biology Laboratory, China Agricultural University. IJs were propagated and collected by EPN infection of *G. mellonella* larvae using the White trap technique. Twenty *G. mellonella* larvae were placed in a plastic Petri dish (90 mm × 15 mm) with a double layer of filter paper, and 2 ml water containing approximately 1,000 IJs of EPN was transferred to the body surface of the *G. mellonella* larvae and incubated at 25°C in the dark for approximately 4 days. The dead *G. mellonella* was transferred to plastic Petri dishes (60 mm × 15 mm) in White traps to collect a large number of IJs drilled from cadavers. The emerged EPNs were washed with sterile water and collected in 250 ml tissue culture flasks at 13–15°C. EPNs stored for less than 2 weeks were used for biological assays (Kaya and Stock, 1997; Glare and Moran-Diez, 2016).

### The effects of IJ density on host mortality

Six densities of IJs were designed to examine the effects of IJ density on host mortality. Virulence assays of EPNs with different densities were performed using last instar larvae of the greater wax moth *G. mellonella*. *G. mellonella* larvae were surface sterilized in 70% (vol/vol) ethanol, washed with sterile water and anesthetized over ice. Approximately 10 μl IJ suspensions of different densities of 5 IJ/larva, 10 IJ/larva, 20 IJ/larva, 30 IJ/larva, 40 IJ/larva and 50 IJ/larva were injected from the third pair of gastropodia into the body cavity using a sterilized microsyringe (Hamilton 1702 RN, 25 μl). Sterile water without EPNs was used as a control. Infected insects were kept in individual wells of 12-well culture plates with a double layer of wet filter paper at 25°C and 70 ± 5% RH with a photoperiod of 14 h light and 10 h dark (14 l:10 D) and checked for mortality every 6 h postinfection (Subkrasae et al., 2022). In each experiment, 10 insects were infected with each density, and each experiment was performed independently at least 5 times.

### Esterase activity assay of *G. mellonella*

The esterase activity was determined by measuring the optical density value (D_600_). PBS buffer (0.45 ml, 0.04 mol/l) was added to 3 ml of α-naphthyl acetate solution (3 × 10^−4^ mol/l), shaken well and allowed to stand at 25°C for 5 min. Then, 50 μl of *G. mellonella* hemolymph was added to the above solution. For 25 min at 25°C, 0.5 ml 10 g/l Fast Blue RR Salt solution-15 g/l SDS (2:5, v/v) was added until a stable teal color appeared (approximately 30 min), the optical density value was determined, and each experiment was performed independently at least 5 times.

### Hemolymph metabonomics of *G. mellonella*

Based on the different levels of mortality caused to the insects by injecting nematodes at different densities, combined with the variable esterase activity in the hemolymph, we chose to perform metabolomic analysis of hemolymphs 6 h after IJ injection, sterile water injection, and without any treatment to prevent the dissociation of fat bodies and the contamination of hemolymphs by other substances. Eleven biological replicates were prepared for the treatment and control groups. The last proleg of the larva was cut with anatomical scissors after a period of treatment, the collected hemolymph was transferred to a 1.5 ml centrifuge tube that had been pretreated in an ice bath, and 50 μl of hemolymph was collected in each repeat. Immediately, 200 μl MeOH-ACN (1:1, v/v) was added to the upper layer of the collected hemolymph, avoiding contact of the hemolymph with air to prevent oxidation, and the mixture was shaken for 60 s to mix well. After centrifugation (centrifuge 5417R, Eppendorf) at 12000 rpm and 4°C for 10 min, all the supernatant was taken and transferred to a new 1.5 ml centrifuge tube, and the solvent was dried using a vacuum concentrator (Concentrator plus, Eppendorf). The dried samples were redissolved in an equal solution of 40 μl MeOH-H_2_O (1:1, v/v) for 30 min by shaking at room temperature. The redissolved solution was centrifuged at 4°C for 10 min at 12000 rpm, and the supernatant was put into a sample bottle for analysis, filtered through a 0.1 μm membrane and transferred to sample vials for UPLC-HRMS analysis. Equal amounts of supernatant from all samples were mixed as QC (quality control) samples for testing. The program of UPLC-HRMS analysis was the same as that in a previous study with some modifications (Cao et al., 2016). The hemolymph extract was analyzed using a UPLC-HRMS system (UPLC, ACQUITY UPLC I-Clas Bio, Waters; MS, Q-Exactive Focus, Thermo Scientific) equipped with a heated electrospray ionization (HESI) source. Positive ion mode was used for MS analysis. The instrument was calibrated using external standards prior to analysis to ensure mass accuracy above 3 ppm throughout the experiment.

### Activity bioassay of potential biomarkers

Differential metabolites were tested against the last instar *G. mellonella* larvae using 12-well culture plates for their insecticidal activity. The compounds were dissolved in sterile water to prepare a 1 mg/ml final solution, and 1 μl of the solution was injected into the body of *G. mellonella* using a microsyringe. The other treatments were the same as those in the above experimental section of the entomopathogenic capacity test.

The corrected mortality of each compound was calculated based on insecticidal biometric data using the following formula:

corrected mortality% = 100 × [(mortality% in treatmentt − mortality% in control)/(100 − mortality% in control)](Bi et al., 2018).

### Statistical analysis

One-way factorial ANOVA was used to analyze the effects of IJ suspensions and different metabolites on the mortality of *G. mellonella*. All analyses were performed using SPSS software 13.0 (SPSS Inc., Chicago, IL, United States). Origin software 2020 (OriginLab Inc., Massachusetts, United States) was used to perform logistic analysis and map the mortality rates of *G. mellonella.*

SIEVE 2.1 software (Thermo Scientific) was used for peak alignment, background subtraction and component extraction of the raw data. SIMCA software 13.0 (Umetrics Inc., Sweden) was used for multivariate statistical analyses and principal component analysis (PCA). HMDB, KEGG, LIPID, MAPS, MassBank, MeSH, METLIN and PubChem were used for metabolite identification to generate a list of candidate chemical formulas. The fragment ion spectra were matched with candidate compounds by MS/MS spectral database matching. Progenesis QI 2.0 (Waters) was used to compare the MS/MS spectra with the theoretical fragmentation pattern. Pathway analysis was performed using MetaboAnalyst 3.0 with high-quality KEGG metabolic pathways as the backend knowledgebase.

According to the MSI guidelines, metabolite identifications can be classified as level II based on their spectral similarity to public/commercial spectral libraries (Sumner et al., 2007; Cao et al., 2016).

## Results

### The infestation of EPNs against *Galleria mellonella*

**Figure 1:**
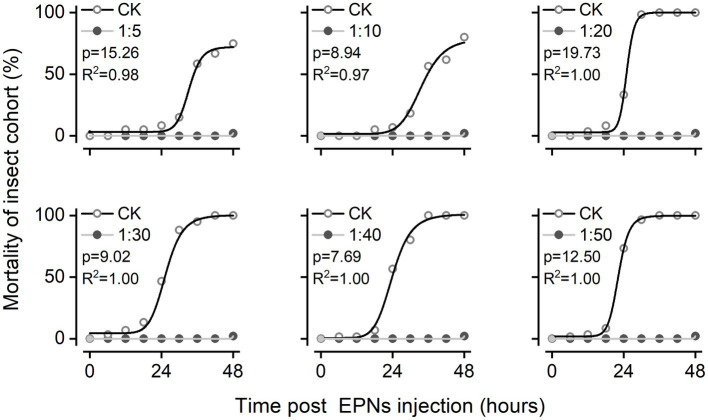
Changes in *G. mellonella* after nematode injection: **(A)** Insecticidal activity of *S. feltiae* injected with different densities against *G. mellonella*. Analysis using the logistic function (y = A2 + (A1 − A2)/(1 + (x/x0)^p)) in nonlinear fitting. **(B)** Apparent changes in the phenotype and esterase activity of *G. mellonella*. After exposure of 20 IJs to the body cavity.

Insecticidal activities at 6 densities of IJs were assessed by hemocoelic injection of freshly grown live *S. feltiae* into last instar larvae of the greater wax moth. All IJ treatments exhibited insecticidal activities. However, their insecticidal activities were different; the mortality of *G. mellonella* was 8.33 ± 0.69, 6.67 ± 0.75, 33.33 ± 1.37, 46.67 ± 2.81, 56.67 ± 2.49, and 73.33 ± 1.49% in 5, 10, 20, 30, 40, and 50 IJs after 24 h (*p* < 0.05). At this time point, the mortality of *G. mellonella* was positively correlated with the amount of IJs but trended toward 100% for all treatments 48 h after injection (Figure 1A).

By observing the changes in *G. mellonella* after injection of 20 IJs, we found that the larval body surface gradually turned gray approximately 12–15 h after injection and then quickly turned black and died in a short time. To characterize the activation of the immune response in *G. mellonella* by *S. feltiae* in more detail, we analyzed esterase activities after the injection of *S. feltiae*. As described before, *S. feltiae* was found to be a strong elicitor of esterase activities, with a maximal induction at 24 h postinjection of approximately 2-fold above the basal level. The induction of esterase activity by IJs was detected at the 9th hour, followed by a slight decrease and a rapid increase until the insect died (Figure 1B). The changes in esterase activities indicated that the autoimmune system was activated to mount an immune response after exposure to IJs, with subsequent rapid destruction of the immune system and fat body hydrolysis leading to a rapid increase in esterase activity.

### Identification, classification and pathway analysis of differential compounds

**Figure 2:**
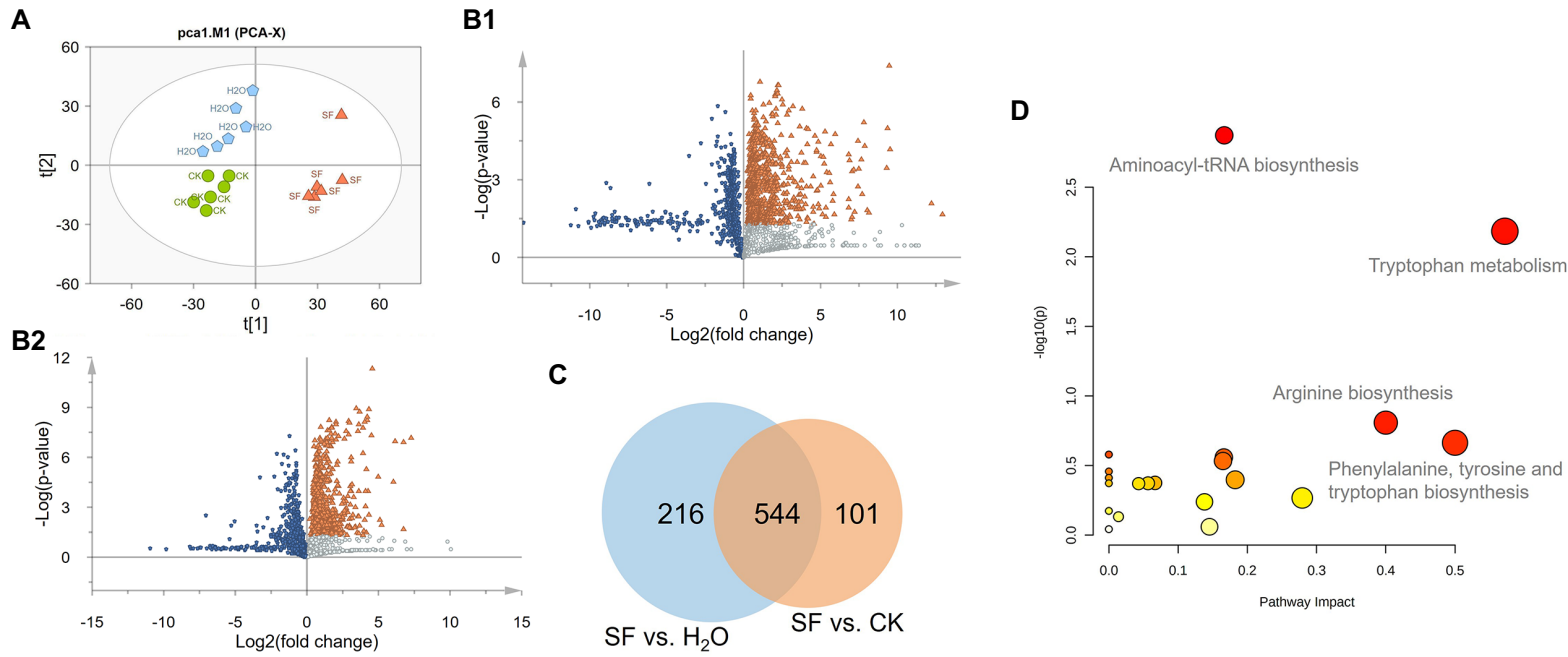
Statistical analysis of the normalized dataset: **(A)** PCA score plot of the 3 treatments. Green indicates CK, blue indicates H_2_O, and orange indicates SF; **(B)** Volcano plots of SF vs. CK and SF vs. H_2_O. Blue represents downregulation, orange represents significant upregulation, and silver represents upregulation but no significant difference. **(C)** Venn diagram of significantly differentially abundant compounds. **(D)** Results of pathway analysis. The pathway impact value was calculated by pathway topology analysis.

A total of 17,144 ion signals (above the 5 × 10^5^ peak intensity threshold) were detected by SIEVE software. The samples with high normalization factors were deleted and focused on metabolites that showed significant variations between different genotypes and treatment groups (ANOVA *p* value <= 0.05, max fold change > = 1.5, and minimum CV < = 20), and 1896 differential features were obtained. PCA was performed on these ion signals to reveal the inherent differences within these signals. The PCA-Score Plot showed that the SF and CK groups were mainly separated along PC1 (34.1%), while PC2 (17.9%) represented a difference between CK and H_2_O. The long distance along both PC1 and PC2 between SF and H_2_O indicated that there were major differences in metabonomics between the two treatments (Figure 2A). Volcano plots showed that 772 compounds in SF vs. CK had Log_2_(fold change) less than 0, and 760 compounds had Log_2_(fold change) greater than 0 and -Log_10_(*p* value) greater than 1.3. The Log_2_(fold change) of 798 compounds in SF vs. H_2_O was less than 0, and the Log_2_(fold change) of 645 compounds was greater than 0 and -Log_10_(*p* value) was greater than 1.3 (Figure 2B). We subsequently performed Venn analysis for those substances that simultaneously satisfied Log_2_(fold change) greater than 0 and -Log_10_(*p* value) greater than 1.3 in the above two group comparisons and found that 544 of these compounds were identical (Figure 2C). Then, the 400 different compounds with the highest VIP value or maximum abundance were selected through high-resolution molecular and fragment ions. According to the metabolite database and theoretical fragmentations, accurate mass, isotopic pattern and MS/MS spectra were searched for secondary identification, and finally, 158 compounds were identified. These compounds included organoheterocyclic compounds, phenylpropanoids and polyketides, benzenoids, organic acids and derivatives, organic oxygen compounds, organosulfur compounds, nucleosides, nucleotides, and analogs. Through pathway enrichment and pathway topology analysis, the 4 most related metabolic pathways were identified, including aminoacyl-tRNA biosynthesis, Trp metabolism, arginine biosynthesis, and phenylalanine, tyrosine and Trp biosynthesis (Figure 2D).

**Figure 3:**
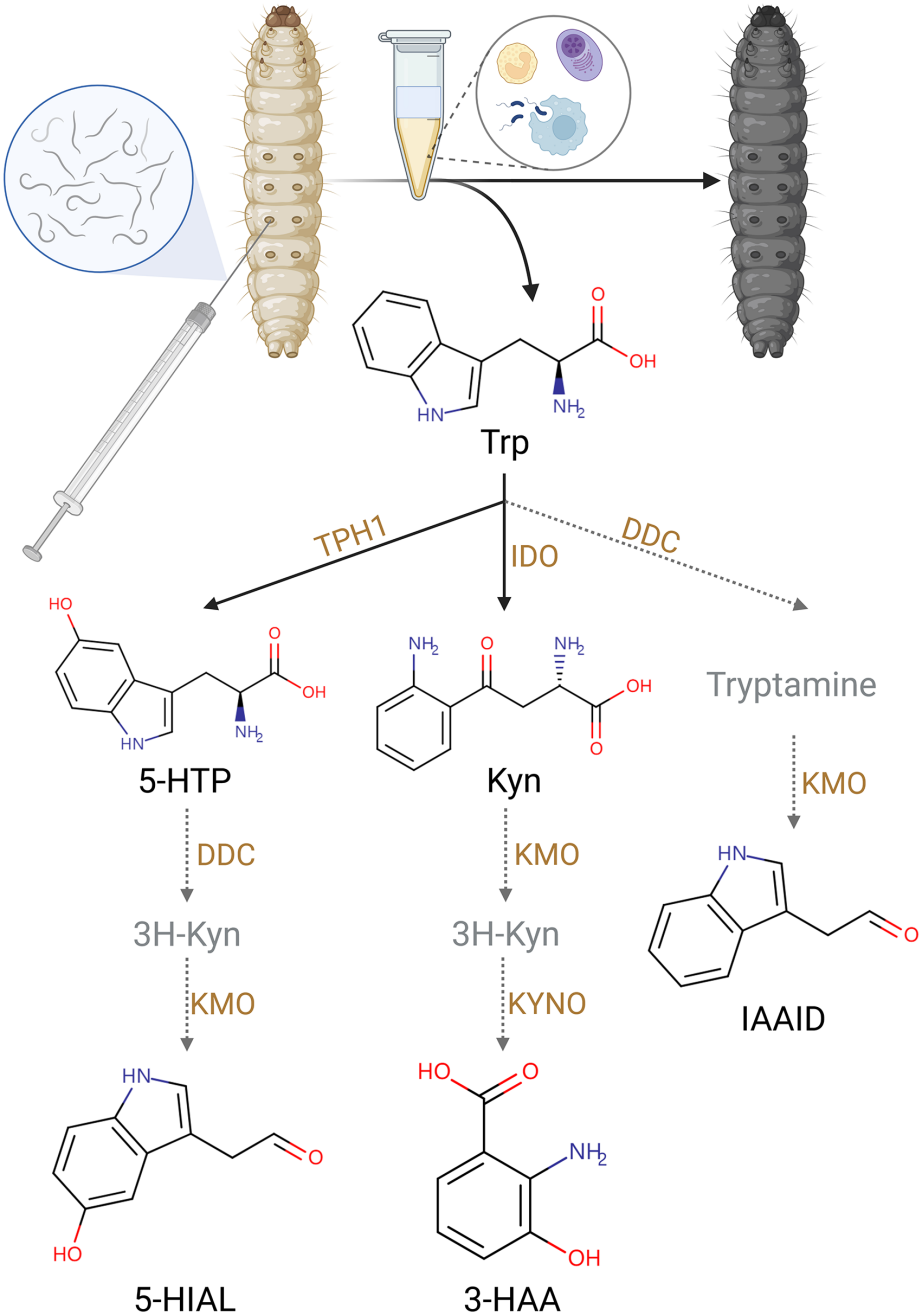
The upregulation of Trp metabolites by injection of *S. feltiae*-*X. bovienii* (Created with BioRender.com).

Trp pathway was impacted by IJ infestation. Trp is the metabolic precursor of many important secondary metabolites. They are essential for organismal growth and development, immune defense, and insect pathogen interactions. Trp, Kyn, 3-HAA, 5-hydroxy-L-tryptophan (5-HTP), 5-hydroxyindoleacetaldehyde (5-HIAL), and indoleacetaldehyde (IAAId) showed higher contents in the SF invasive group. The changes in these metabolites suggested exposure to *S. feltiae*-*X. bovienii* to the hemocoel of *G. mellonella* activated the Trp pathway and especially the Kyn pathway (Figure 3).

### Determination of insecticidal biological activity

**Figure 4:**
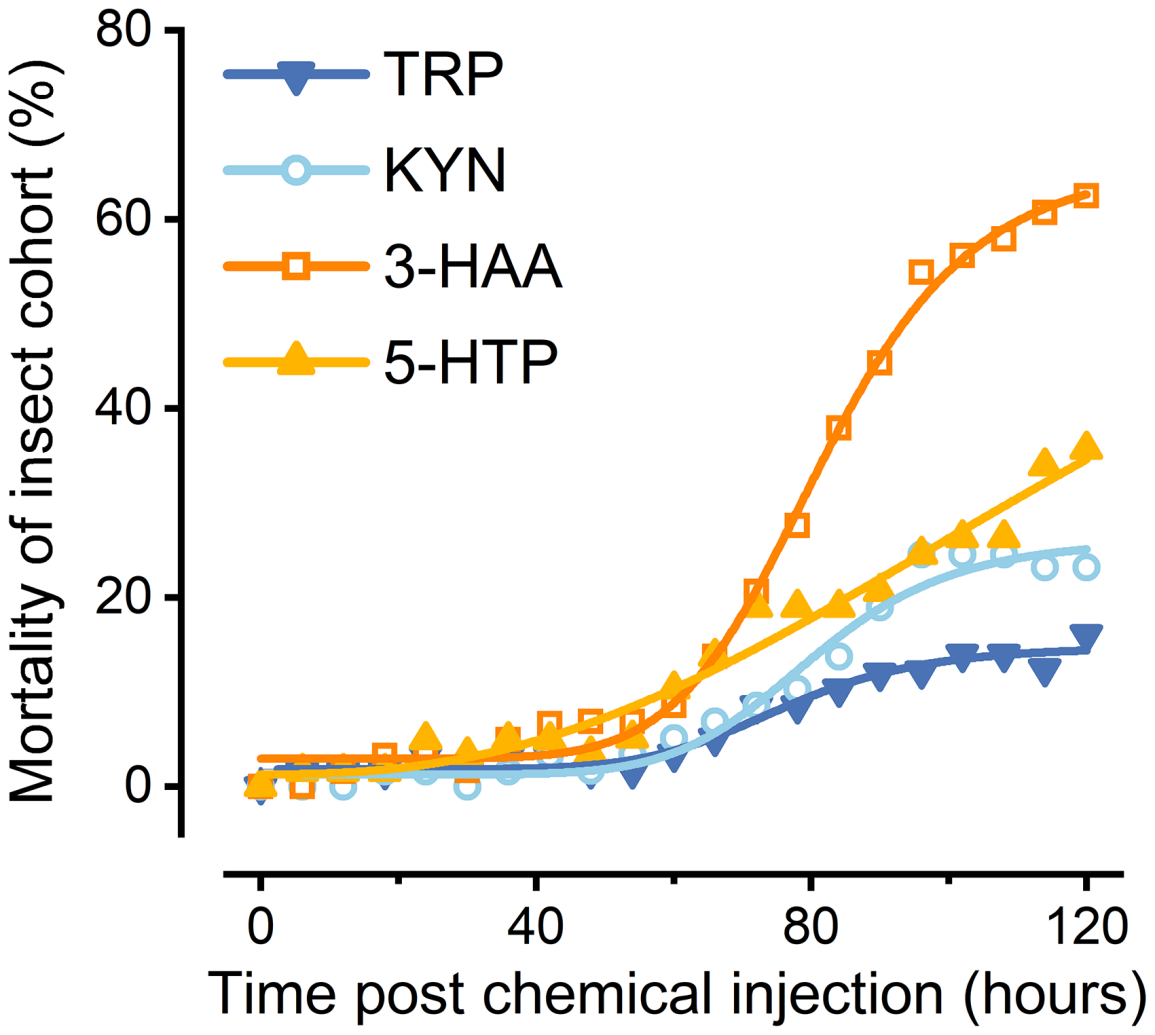
Preliminary insecticidal activity of Trp metabolites.

All the evaluated compounds (Trp, Kyn, 3-HAA, and 5-HTP) demonstrated insecticidal activities with variations between compounds (Figure 4). Trp also caused the death of the great wax borer, although it exhibited much lower insecticidal viability than several other compounds. The compound that showed the highest insecticidal activity was 3-HAA. In contrast, Kyn and 5-HTP had relatively low toxicities to *G. mellonella*.

## Discussion

Compound infestation with EPNs and commensal bacteria can cause insects to die with great efficiency, but insects also undergo a series of immune responses before dying to fight the self-inflicted injury from the invasion of exosomes, such as producing an array of esterases to enhance autoimmunity (Bhatt et al., 2020). Our study demonstrated that esterase activity in the body increased initially and then decreased after nematode infestation. It was hypothesized that an earlier increase in esterase activity occurred when the insect identified the nematode infestation on its own and thus synthesized more esterases to clear the foreign interference, while the subsequent decline in activity was brought on by the release of commensal bacteria that had damaged the insect immune system, leaving the insects without the ability to fight off infection. The host insect’s immune system gradually disintegrated over time, and the fat body, which served as the hub of the insect immune system, was also destroyed. This led to a persistent increase in esterase activity in the hemolymph.

Trp metabolism is crucial for pathogen and host microbiota interactions. Although there has been relatively little research in the field of insects, it has recently been found that in tripartite *Xenorhabdus* bacterium-*Steinernema* nematode-*Galleria* insect symbiosis, the Trp metabolism pathway, especially the Kyn pathway, plays important functions at different stages (Mucci et al., 2022). We discovered that after *S. feltiae*-*X. bovienii* were injected into the hemocoel of *G. mellonella*, the Trp metabolic pathway of the insects was significantly upregulated, especially the 3-HAA content of the Kyn pathway, which was significantly increased in the treated group. It was verified that the injection of 1 mg/ml 3-HAA into *G. mellonella* could cause metabolic disorder or disease and ultimately cause the death of the host. These findings provide direct evidence that Trp metabolites kill *G. mellonella* and that Trp metabolism is crucial for controlling how nematodes, symbiotic bacteria, and insects interact.

Previous research has demonstrated that the excreted/secreted products produced during the infection process can alter the host’s immune response (Duarte et al., 2013; Chang et al., 2019). Numerous Trp metabolites have regulatory effects on immune cells, 3-HAA at appropriate concentrations has anti-inflammatory activity (Lee et al., 2016), and significant upregulation of 3-HAA has been identified in a rat brain injury model (Mangas et al., 2018). 3-HAA has also been identified as a crucial molecule for vascular Trp regulation in inflammation and lipid metabolism (Martin et al., 2019). Although anthranilate was able to affect the biofilm formation, virulence, and antibiotic tolerance of *Pseudomonas aeruginosa* (Kim et al., 2015; Hwang et al., 2022), our experiment revealed that high concentrations of 3-HAA were not favorable for its survival. *Xenorhabdus* have been reported to utilize the fatty acids of host insects to synthesize some natural products (Proschak et al., 2011; Shi and Bode, 2018). Many selective toxic secondary metabolites produced by entomopathogenic bacteria are associated with phenylethylamides and tryptamides (Bode et al., 2017; Tse et al., 2017; Kusakabe et al., 2022; Lefoulon et al., 2022). For example, *X. doucetiae* DSM179 can synthesize large quantities of these related substances (Bode et al., 2017), which can modulate quorum sensing in other bacteria and partially exhibit quorum quenching activity (Morohoshi et al., 2008; Lang and Faure, 2014; Johnson et al., 2016). In other bacteria, *Klebsiella oxytoca* produces tilimycin or kleboxymycin, which can attack imine intermediates nonenzymatically, causing cytotoxicity 5-fold higher than that of tilivalline (Dornisch et al., 2017; Tse et al., 2017), and *Mycobacterium tuberculosis* escapes the host’s defense response by synthesizing Trp (Zhang et al., 2013). However, whether *X. bovienii* can synthesize some substances with insecticidal activity using Trp or Trp metabolites produced by the host insect is not known and is a matter of great interest in pathogenic organisms, microorganisms and insect interactions.

## Conclusion

In this study, metabonomics revealed the Trp metabolic pathway as the most predominant pathway in the context of host pathogenicity, with 3-HAA being the most likely to mediate EPN killing of host insect potential effectors. The Trp, 5-HTP, KYN, and 3-HAA levels in the hemolymph of *G. mellonella* larvae changed significantly due to infection with the EPN *S. feltiae* and its gut symbiont *X. bovienii.* We also demonstrated that EPNs at very low densities can cause a great degree of death in host insects when they reach the host body. Furthermore, the upregulation of esterase activity in the hemolymph of the host was determined to be necessary for microbial-mediated defenses, which is the result of specific effects of EPN infection. These findings provide a reference for deeply mining the biocontrol potential and analyzing the pathogenic mechanisms of EPNs and provide an important basis for the development of biopesticides, which can even be used in the medical and health care industries, helping to advance the understanding and research of human parasitic diseases.

## Data availability statement

The raw data supporting the conclusions of this article will be made available by the authors, without undue reservation.

## Author contributions

YZ completed the trial, collected data, and wrote original draft manuscript. ZZ and FW designed the study and supervised the project. All authors reviewed, edited the manuscript and approved the final version.

## Funding

This research is supported by the National Key R&D Program (No. 2021YFC2600401) and the Science and Technology Key Project from Yunnan branch of China Tobacco Corp oration (2022530000241021).

## Conflict of interest

The authors declare that the research was conducted in the absence of any commercial or financial relationships that could be construed as a potential conflict of interest.

## Publisher’s note

All claims expressed in this article are solely those of the authors and do not necessarily represent those of their affiliated organizations, or those of the publisher, the editors and the reviewers. Any product that may be evaluated in this article, or claim that may be made by its manufacturer, is not guaranteed or endorsed by the publisher.

## Abbreviations

EPN: Entomopathogenic nematode
IAA: Indole-3-acid-acetic
IAld: Indole-3-aldehyde
IAAId: Indoleacetaldehyde
IJ: Infective juvenile
Kyn: Kynurenine
PCA: Principal component analysis
QC: Quality control
Trp: Tryptophan
TpH 1: Tryptophan hydroxylase 1
3-HAA: 3-hydroxyanthranilic acid
5-HIAL: 5-hydroxyindoleacetaldehyde
5-HTP: 5-hydroxy-L-tryptophan.
